# In Situ Second Harmonic Generation and Extinction Spectroscopy for Studying Colloidal Gold–Silver–Gold Core–Shell–Shell Nanoparticle Growth Dynamics

**DOI:** 10.1021/acs.jpcc.5c02596

**Published:** 2025-06-13

**Authors:** Stena C. Peterson, Daniel A. Babayode, Christopher P. Reso, Louis H. Haber

**Affiliations:** Department of Chemistry, 5779 Louisiana State University, Baton Rouge, Louisiana 70803, United States

## Abstract

Time-dependent in situ second harmonic generation (SHG) spectroscopy coupled with extinction spectroscopy is used to monitor the growth dynamics involved in the synthesis of colloidal gold–silver–gold core–shell–shell (Au–Ag–Au CSS) nanoparticles in real time. A stepwise seed-mediated method is applied to grow an outer gold shell onto gold–silver core–shell nanoparticles in aqueous solution, using four sequential additions of chloroauric acid and reducing agents. The first addition results in Au–Ag–Au CSS nanoparticles with a bumpy, urchin-like morphology. With each subsequent addition, the outer gold shell thickness increases, while the nanoparticle surface morphology becomes smoother and more uniform. Transmission electron microscopy (TEM) is also utilized to determine the nanoparticle size distribution and surface morphology after each addition. As the size and surface smoothness of the CSS nanoparticles increase, the plasmon extinction spectra blue shift with spectral narrowing and increasing extinction intensity. Comparison with corresponding Mie theory extinction spectra calculations shows general agreement after the fourth addition, demonstrating a spherical concentric nanoarchitecture with a smooth nanoparticle surface. The surface-sensitive SHG signal increases dramatically during the first addition, corresponding to the urchin-like surface morphology, and then decreases as the surface becomes smoother with each subsequent addition. In situ monitoring of the two-photon fluorescence (TPF) signal provides complementary information for comparison to the extinction and SHG results. This combined approach of in situ SHG and extinction spectroscopy with Mie theory simulations and TEM imaging provides a detailed analysis of the synthesis of Au–Ag–Au CSS nanoparticles for investigating complex colloidal nanoparticle growth dynamics occurring at the nanoscale.

## Graphical abstract

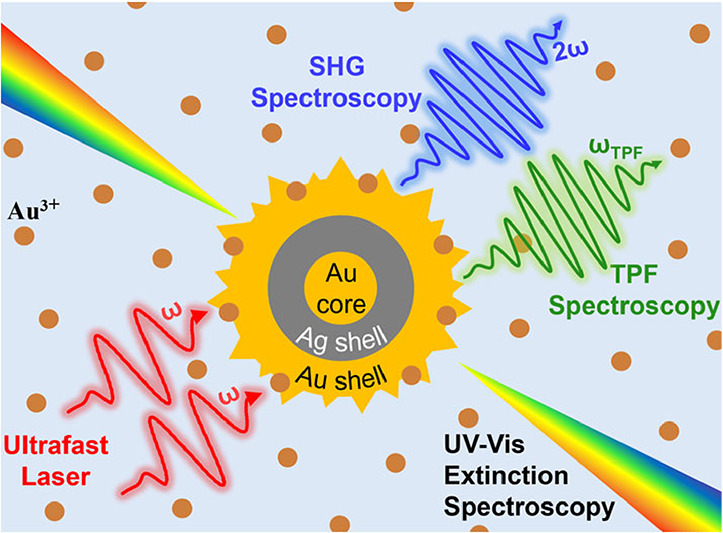

## Introduction

Plasmonic nanoparticles are of great interest for a wide variety of applications, especially in the biomedical field. Noble metal nanoparticles of gold and silver possess unique optical, electronic, catalytic, and chemical properties which differ from those of their bulk constituents,^1–4^ where enhanced optical absorption and scattering processes arise from the localized surface plasmonic resonances (LSPRs) due to collective oscillations of free electrons near the metal nanoparticle surface at resonant incident optical frequencies.^5–13^ The plasmonic spectra depend on the size, shape, composition, surface morphology, and surrounding medium of the nanoparticles, providing a tunability of the nanomaterial optical properties.^1,2,4,5,8,14–18^ Monometallic and bimetallic nanoparticles composed from gold and silver are excellent candidates for nanomedical applications due to their wavelength-dependent dielectric properties, the ease of synthesis, chemical biocompatibility, and tunable LSPRs covering the visible and near-infrared (NIR) optical regions.^2,4,8,9,19^ Additionally, with core–shell and core–shell–shell nanoparticles, the plasmon spectra can be controlled and optimized by varying the size of the nanoparticles as well as the ratio of core to shell thickness.^1,4^ These plasmonic nanoparticles are specifically advantageous for nanomedicinal technologies, including photothermal therapy, biosensing, drug delivery, molecular sensing, and gene therapy.^4,8,20–24^

Core–shell and core–shell–shell noble metal nanoparticles are leading candidates for these developing biomedical nanotechnologies due to their tunable optical properties, especially in the visible and NIR regions,^25–27^ although more research is needed to optimize their size-dependent plasmonic photothermal efficiencies according to the desired applications. Photothermal therapy is a specialized cancer treatment method that utilizes photothermal agents and incident optical irradiation to induce localized hyperthermia in cancerous cells and tumors, which are typically heated to a temperature of 41 to 45 °C, while leaving healthy cells and tissue largely unharmed.^18,20–22,28,29^ Noble metal nanoparticles are ideal photothermal agents due to their exceptionally strong optical absorption from LSPRs in the visible and NIR range, leading to very high photothermal efficiencies, especially for nanomaterials containing gold.^15,30–34^ These nanoparticle surfaces can also be functionalized with biomolecules and drug molecules for selective biological targeting and drug-delivery applications.^34–36^

There is a wide variety of analytical techniques used for the characterization of plasmonic nanoparticles, including UV–vis extinction spectroscopy, dynamic light scattering (DLS), transmission electron microscopy, X-ray diffraction (XRD), X-ray photoelectron spectroscopy (XPS), atomic force microscopy (AFM), and small-angle X-ray scattering (SAXS).^19,37–39^ These techniques are extensively utilized for ex situ characterization, after the nanoparticle synthesis has completed. However, in situ characterization methods are needed to monitor the growth mechanisms and chemical reactions occurring during the nanoparticle synthesis in real-time for investigating nanomaterial engineering to optimize these procedures while developing more complicated nanoarchitectures such as core–shell and core–shell–shell nanoparticles with controlled surface morphologies. In our previous work, in situ SHG spectroscopy coupled with in situ extinction spectroscopy was used to study the growth dynamics and nanomaterial chemical reactions involved in the synthesis of gold nanoparticles, gold–silver core–shell nanoparticles, and silver–gold core–shell (Ag–Au CS) nanoparticles.^19,30,40,41^ Real-time monitoring of the syntheses of these plasmonic nanoparticles demonstrated differences in the amount of time required for the size and the surface morphology to reach the corresponding equilibria in these different chemical and nanomaterial-based reactions in aqueous colloidal suspension.

Second harmonic generation spectroscopy is a powerful nonlinear optical technique for the study of surfaces, interfaces, and other noncentrosymmetric structures.^42–52^ In SHG, the coherent addition of two incident photons with frequency ω combine to produce a photon with frequency 2ω in a process which is dipole forbidden in centrosymmetric bulk media.^19,53–57^ The inversion symmetry is broken at the surface of colloidal nanoparticles, allowing for surface-sensitive SHG signals.^45,48,58,59^ The SHG signal from a colloidal nanoparticle sample depends on several factors including the nanoparticle size, nanoparticle spectroscopy, surface morphology, surface chemistry, and electrostatic surface potential.^19,30,40,41^ Because the SHG signal predominately originates from the nanoparticle surface, time-dependent in situ SHG studies are ideal for investigating the surface-sensitive chemical and structural changes occurring during the nanoparticle growth reactions.^19,55,60^ Nanoparticle surface reactions and corresponding structural changes, such as the formation and evolution of plasmonic hotspots, can be investigated during the nanoparticle synthesis using these time-dependent SHG measurements.^5,61–63^

Two photon fluorescence is another type of nonlinear optical process which involves the simultaneous absorption of two incident photons of frequency ω, leading to an excited-state transition and subsequent relaxation, followed by the emission of a photon with frequency ω_TPF_ that is less than 2ω.^64–66^ LSPRs are known to significantly enhance nonlinear optical processes, including SHG, two-photon absorption, and TPF signals.^30,67–69^ Many studies have demonstrated that TPF and two-photon photoluminescence (TPPL) properties of noble metal nanoparticles strongly depend on the nanoparticle shape, surface morphology, and corresponding LSPR spectrum.^70–72^ A recent study on the shape-dependence of TPPL from different types of gold nanoparticles reported a huge enhancement (∼50,000 times) from gold nanobranch nanoparticles compared to gold nanospheres, due to the sharp tips and plasmonic hotspots.^73^ TPF also has different symmetry restrictions than SHG, making it less surface-sensitive than SHG, especially for centrosymmetric crystals and nanomaterials such as gold and silver, but more surface-sensitive than extinction spectroscopy.^41^ Therefore, the TPF measurements provide for complementary information regarding changes in size, surface morphology, and nanostructure during plasmonic nanoparticle growth dynamics for direct comparisons to extinction and SHG spectroscopy results. However, more experimental and theoretical research is needed to better understand the correlations between these different nonlinear optical and plasmonic properties.

In this paper, the growth dynamics of gold–silver–gold core–shell–shell nanoparticles are monitored in real time during the stepwise synthesis using in situ SHG spectroscopy coupled with extinction spectroscopy. This is the first time this technique has been used to study a core–shell–shell system, to our knowledge. Here, an outer gold shell is added to colloidal Au–Ag CS nanoparticles with four sequential additions of chloroauric acid, sodium citrate, and hydroquinone in aqueous solution. Transmission electron microscopy is used to determine the final size and surface morphology of the Au–Ag–Au CSS nanoparticles after each addition. An urchin-like surface morphology appears after the first addition, followed by the surface becoming smoother and more uniform as the outer gold shell becomes thicker with the subsequent second, third, and fourth additions. The associated extinction, SHG, and TPF growth lifetimes for each addition are determined by analysis of the in situ spectroscopy results. In situ extinction spectroscopy shows an increase in intensity, blue-shifting, and spectral narrowing as the size of the Au–Ag–Au CSS nanoparticles increases over the course of the stepwise synthesis, with excellent spectral agreement with Mie theory calculations for the final CSS sample. The in situ SHG signal is highest during the first addition, caused predominantly by plasmonic hotspots, followed by the SHG intensity decreasing as the surface morphology becomes more smooth during the second, third, and fourth additions. Analysis of the TPF signal during the nanoparticle growth process provides complementary information about the size and surface morphology of the Au–Ag–Au CSS nanoparticles. Using experimental in situ SHG, TPF, and extinction spectroscopy in real time, combined with TEM and Mie theory calculations, allows for a detailed study of the growth dynamics, nanoparticle size distributions, and surface morphologies involved during the synthesis of Au–Ag–Au CSS nanoparticles for developing advanced colloidal hybrid-plasmonic nanoengineering applications.

## Experimental Section

### Nanoparticle Synthesis and Characterization

Chloroauric acid from Alfa Aesar, sodium citrate and ascorbic acid from Acros Organics, hydroquinone and silver nitrate from Thermo Scientific, and sodium hydroxide from VWR are used in ultrapure water without further purification. First, gold nanoparticle seeds are prepared for the gold core^25,40^ where 900 μL of 34 mM sodium citrate is added to a vigorously stirring, boiling solution of 30 mL of 290 μM chloroauric acid in ultrapure water. The colloidal solution is refluxed for 20 min under vigorous stirring during which the solution undergoes a color change from pale yellow to bright red and is then cooled to room temperature. Next, a silver shell is grown onto the core gold seeds to prepare gold–silver core–shell nanoparticles^25^ using 300 μL of the previously prepared gold seeds added to 10 mL of ultrapure water at room temperature. Then, 15 μL of 100 mM silver nitrate, 60 μL of 100 mM ascorbic acid, and 75 μL of 100 mM sodium hydroxide are added to the Au seed solution, which is vigorously stirred for 30 min at room temperature, with the color changing to a light brownish yellow indicating the formation of Au–Ag CS nanoparticles.

Finally, gold–silver–gold core–shell–shell nanoparticles are prepared through a seed-mediated growth approach^19,25,40,41^ which utilizes a stepwise reduction of chloroauric acid onto the Au–Ag CS nanoparticles. The thickness of the outer gold shell can be controlled by varying the concentrations of the Au–Ag CS nanoparticles, chloroauric acid, sodium citrate, and hydroquinone used.^19,41,74^ Here, four sequential additions of chloroauric acid and reducing agents are performed in a quartz cuvette. For each addition, the growth process of the gold shell at the surface of the nanoparticles is initiated by the addition of the reducing agent mixture, which is composed of 11 μL of 7.7 mM sodium citrate and 23.2 mM hydroquinone. For the first addition, 4.3 μL of 25 mM chloroauric acid is added to an aqueous solution of 15 μL of the previously prepared Au–Ag CS NPs and 2.5 mL of ultrapure water, followed quickly by the addition of the 11 μL reducing agent mixture. For the second, third, and fourth additions, 8.5, 12.5, and 4.3 μL of 25 mM chloroauric acid are added, respectively, to the aqueous solution simultaneously along with the reducing agent mixture. The colloidal nanoparticle solution is constantly stirred at room temperature while undergoing in situ SHG and extinction spectroscopic measurements to monitor the growth dynamics in real time. The first, second, and third additions are monitored sequentially for 1360 s each, then the fourth addition is monitored for 4090 s.

TEM images of the nanoparticles are obtained using a JEOL-1400 microscope with carbon-coated copper grids for determining the nanoparticle morphologies and size distributions after each addition. Additional characterization results on the Au seeds, Au–Ag CS nanoparticles, and Au–Ag–Au CSS nanoparticles are provided in the Supporting Information. The final CSS nanoparticles are also characterized with TEM and extinction spectroscopy after washing twice, where 1.0 mL of the final colloidal CSS nanoparticle solution is centrifuged at 2400 rpm for 10 min then redispersed in 135 μM sodium citrate in 1.0 mL of ultrapure water.

### In Situ Second Harmonic Generation and Extinction Spectroscopy

The combined time-dependent in situ second harmonic generation spectroscopy and in situ extinction spectroscopy setup has been described in our previous studies.^19,30,40,41^ The SHG setup consists of a titanium:sapphire oscillator laser with the wavelength centered at 800 nm, a 75 fs pulse width at a repetition rate of 80 MHz, and an average output power of 1.0 W. The laser beam is attenuated to 400 mW using a neutral density filter and is then focused into a 1 cm by 1 cm quartz cuvette containing the aqueous colloidal nanoparticle solution. A high-sensitivity spectroscopy charge-coupled device (CCD) detector connected to a monochromator spectrograph collects the SHG and TPF signals as a function of time in the forward direction. The extinction spectroscopy setup consists of a tungsten filament lamp that generates a low-intensity, broadband white light beam which passes through the quartz cuvette and nanoparticle sample orthogonal to the SHG beam. A fiber optic connected to a spectrometer detector measures the in situ extinction spectra. The growth of the outer gold shell on the Au–Ag CS nanoparticles is initiated by the addition of the reducing agent mixture to the solution of Au–Ag CS nanoparticles, chloroauric acid, and water, which is designated as time zero for the first addition. This is followed by the subsequent additions of chloroauric acid and reducing agents to the colloidal nanoparticle solution for the second, third, and fourth additions.

## Results and Discussion

Transmission electron microscopy is used for the characterization of the Au–Ag–Au CSS nanoparticles produced after each addition of chloroauric acid and reducing agents. The average nanoparticle diameter for each addition is determined from the log-normal fit of the size distribution histogram, which is constructed from measurements of over 150 nanoparticles, as shown in the Supporting Information. The average nanoparticle sizes for the Au–Ag–Au CSS nanoparticles after the first, second, third, and fourth additions of chloroauric acid and reducing agents are 38 ± 11, 48 ± 12, 62 ± 17, and 80 ± 22 nm, respectively. The Au–Ag–Au CSS nanoparticles have an inner Au–Ag CS diameter of 21 ± 7 nm with a gold core diameter of 13 ± 2 nm, as determined by TEM measurements of the Au core seeds and Au–Ag CS NP samples used.

Representative TEM images of the Au–Ag–Au CSS nanoparticles after each addition of chloroauric acid and reducing agents are shown in Figure 1. The reduction of chloroauric acid onto the surface of the Au–Ag CS nanoparticles after the first addition results in a rough outer gold shell with a bumpy, urchin-like Au–Ag–Au CSS nanoparticle surface morphology, as seen in Figure 1a. After the second addition, in Figure 1b, the thickness of the outer gold shell increases while the surface morphology of the Au–Ag–Au CSS nanoparticles becomes slightly smoother than after the first addition. The size of the Au–Ag–Au CSS nanoparticles increase again after both the third and fourth additions, and the nanoparticles show further smoothening of the nanoparticle surface in Figure 1c,d. However, there are additional small features in the background and superimposed on the nanoparticles in the TEM images of Figure 1c,d, which are attributed to an excess of crystallized salts and reactants that form as the aqueous solution dries on the TEM grids and distort the nanoparticle surface morphologies for these additions. Subsequent studies are performed where the CSS nanoparticle sample is washed to remove excess salts and reactants, as described later. Additional TEM images for the Au seeds, Au–Ag CS nanoparticles, and Au–Ag–Au CSS nanoparticles are also shown in the Supporting Information.

**Figure 1:**
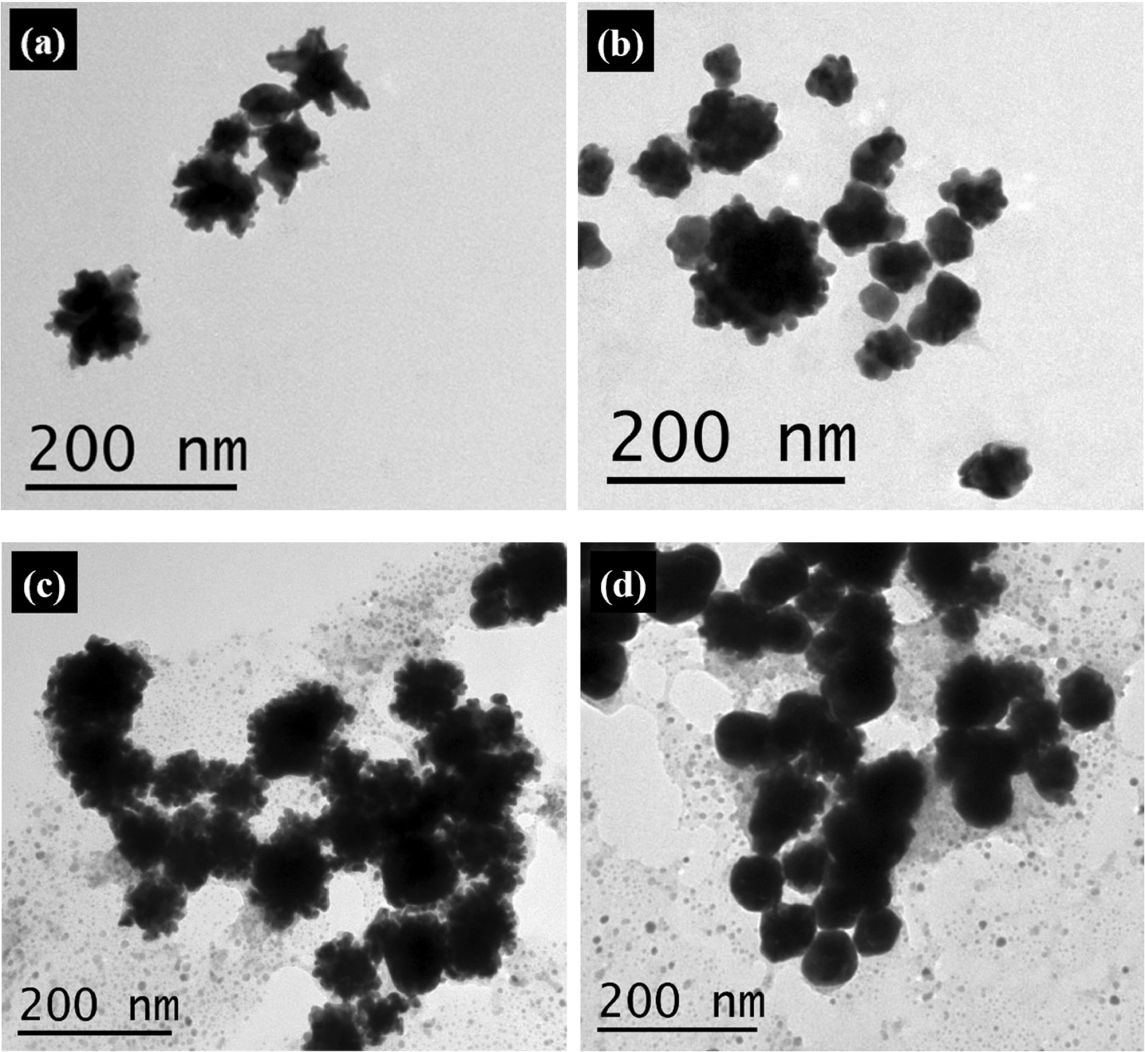
Representative TEM images of Au–Ag–Au CSS nanoparticles after the (a) first, (b) second, (c) third, and (d) fourth additions of chloroauric acid and reducing agents with average sizes of 38 ± 11, 48 ± 12, 62 ± 17, and 80 ± 22 nm, respectively.

In situ SHG spectroscopy coupled with in situ extinction spectroscopy provides additional time-dependent measurements for a detailed analysis of the Au–Ag–Au CSS nanoparticle growth dynamics. The final extinction spectra of Au–Ag–Au CSS nanoparticles after each addition of chloroauric acid and reducing agents are shown in Figure 2 at reaction times of 1310, 2675, 4035, and 8140 s, corresponding to the results after the first, second, third, and fourth additions, respectively. The broad, red-shifted plasmon peak near 577 nm after the first addition of reducing agents corresponds to the urchin-like Au–Ag–Au CSS nanoparticles observed in Figure 1a. This broad plasmonic spectrum is influenced by the sharp points on the nanoparticle surface which create strong localized electromagnetic field enhancements known as plasmonic hotspots, leading to red shifting of the plasmon peak.^5,61–63,75,76^ After each subsequent addition of chloroauric acid and reducing agents, the spectral width of the plasmon peak becomes increasingly more narrow while blue-shifting to 567, 561, and 555 nm after the second, third, and fourth additions, respectively, corresponding to the surface of the Au–Ag–Au CSS nanoparticles becoming smoother after each addition. The increasing Au–Ag–Au CSS nanoparticle size after each addition also causes the observed increase in the plasmon peak intensity.^19,69,73^ Additional information on the change in extinction spectra during the Au–Ag–Au CSS nanoparticles synthesis is provided in the Supporting Information.

**Figure 2:**
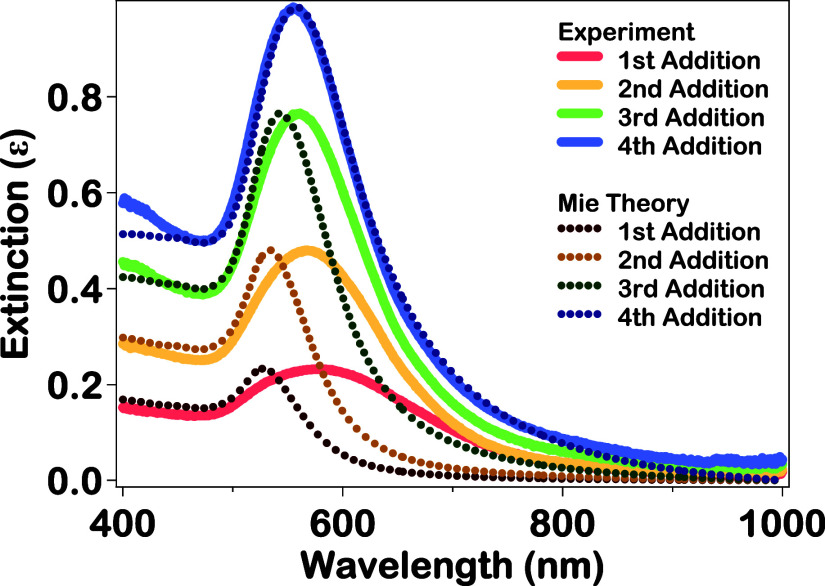
Final extinction spectra of Au–Ag–Au CSS nanoparticles (solid lines) after the first, second, third, and fourth additions of chloroauric acid and reducing agents along with the corresponding Mie theory results for each addition (dotted lines). The large deviations between the experimental and theoretical results for the first, second, and third additions are primarily due to the urchin-like morphology of the nanoparticles. Excellent agreement between the experimental and theoretical results after the fourth addition demonstrates a smoother final CSS nanoparticle surface and a more ideal nanoarchitecture.

The corresponding theoretical extinction spectra from Mie theory calculations of the Au–Ag–Au CSS nanoparticles after each addition are given by the dotted lines in Figure 2. Mie theory is an analytical solution to Maxwell’s equations, allowing for the determination of the scattering and absorption spectra of spherical, core–shell, and core–shell–shell plasmonic nanoparticles.^14^ These spectra are simulated using the Mie Lab program by entering the CSS dimensions, including the diameter of the gold core, the thicknesses of the silver shell and outer gold shell, as well as the standard deviations of these dimensions, which are obtained from the TEM measurements. The optical properties of the surrounding water are also included in the Mie Lab calculations, which assume an ideal, concentric, and spherical CSS nanoarchitecture.^41,77^ Direct comparison of the experimental extinction spectra with the Mie theory calculations provides insight on the size distributions, compositions, and morphologies of the nanoparticles.^30,32^ The Mie theory calculated plasmon peak after the first, second, third, and fourth additions are centered at 528, 533, 543, and 559 nm, respectively. The experimental results are significantly red-shifted in comparison to the Mie theory results for the first, second, and third additions. The deviations between the experimental and theoretical extinction spectra for the first, second, and third additions are primarily caused by the rough, bumpy nanoparticle surface which deviates from ideal CSS architecture, especially due to the plasmonic hot spots at the sharp points of the urchin-like surface morphology.^5,8,19,30,41,76,77^ For the fourth addition, the experimental and theoretical extinction spectra are in excellent agreement in terms of the plasmon peak wavelength and spectral width, demonstrating the high correlation between Mie theory and experiment for these spherical plasmonic Au–Ag–Au CSS nanoparticles with smooth surface morphologies.

The extinction peak maximum time trace is shown in Figure 3 as solid lines, along with the corresponding fits for each addition as dotted lines. The extinction peak maximum values initially increase rapidly after each addition of chloroauric acid and reducing agents, followed by a period of slower change before an equilibrium is reached. The extinction growth lifetimes are determined by fitting the slower change portion of each extinction peak maximum time trace using a single-exponential function, given by ε­(*t*) = *A*_ext_e^–*t*/τ_ext_^ + *B*_ext_ where *A*_ext_ is the extinction peak amplitude, τ_ext_ is the extinction growth lifetime, *B*_ext_ is the offset extinction peak value, and *t* is the reaction time after the addition of reducing agents. As listed in Table 1, the extinction growth lifetimes determined for the first, second, and third additions are 7.0 ± 0.2, 37.5 ± 1.5, and 40.4 ± 0.1 s, respectively. For the fourth addition, the peak maximum values are observed to slowly rise linearly with a slope of (1.98 ± 0.02) × 10^–6^ ε/s after the initial rapid increase, so this extinction growth lifetime is not obtained. The extinction growth lifetimes determined for the first three additions demonstrate that a longer amount of time is required to reach an equilibrium outer gold shell thickness with each subsequent addition. In the Supporting Information, separate extinction peak maximum time traces with the corresponding fits are given in Figure S12 and these fit parameters for each addition are listed in Table S1.

**Figure 3:**
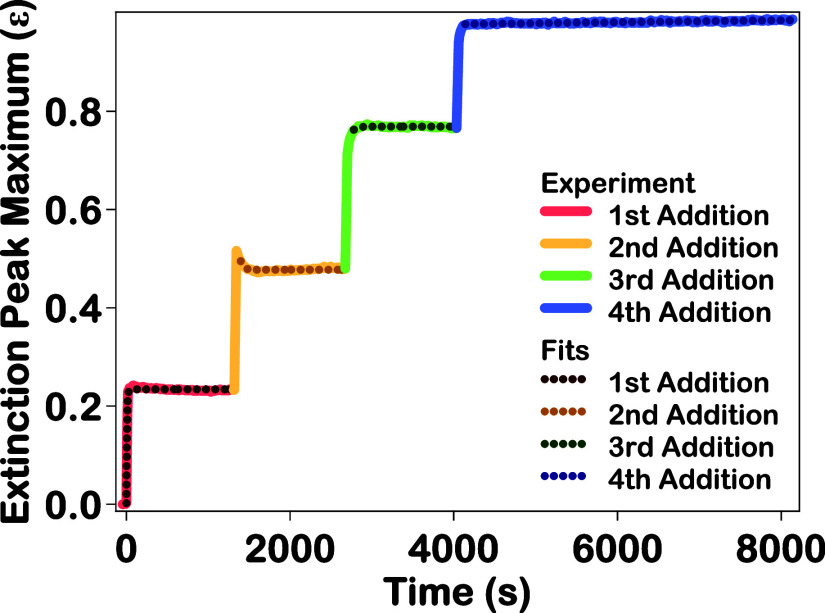
Extinction peak maximum as a function of time for the four stepwise additions of chloroauric acid and reducing agents during the synthesis of Au–Ag–Au CSS nanoparticles (solid lines), along with the corresponding fits (dotted lines).

**Table 1:** Final Sizes of the Au–Ag–Au CSS Nanoparticles after Each Addition with the Corresponding Extinction, SHG, and TPF Growth Lifetimes Obtained from the In Situ SHG and Extinction Spectroscopy of the Four Stepwise Additions in the CSS Nanoparticle Synthesis

addition	final size (nm)	extinction growth lifetime τ_ext_ (s)	SHG growth lifetime τ_SHG_ (s)	TPF growth lifetime τ_TPF_ (s)
first	38 ± 11	7 ± 0.2	126 ± 3	94 ± 2
second	48 ± 12	37 ± 1.5	620 ± 1.5	612 ± 11
third	62 ± 17	40 ± 0.1	681 ± 7	276 ± 44
fourth	80 ± 22		2130 ± 41	

The in situ SHG intensity spectra at various reaction times during the four-addition synthesis of the Au–Ag–Au CSS nanoparticles are shown in Figure 4 to provide complementary information on the growth dynamics. The initial SHG signal is extremely low before the growth of the outer gold shell is initiated, corresponding to the smaller, colloidal Au–Ag CS nanoparticles in water. After the first addition of reducing agents, a SHG peak centered at 400 nm emerges and the signal intensity rapidly increases after the first addition followed by a gradual decrease until an equilibrium is reached. After each subsequent addition of chloroauric acid and reducing agents, there is a relatively rapid increase and decrease in SHG signal intensity, followed by a more gradual decrease in signal over time before a new equilibrium is reached. The SHG signal intensity depends on many factors including the size and surface morphology of the nanoparticles.^5,55,60^ The urchin-like Au–Ag–Au CSS nanoparticle surface after the first addition corresponds to the highest SHG signal intensity, resulting from the strong enhancement due to the plasmonic hotspots created by the sharp points on the urchin-like surface morphology.^74,76^ A general decrease in SHG signal intensity through the remainder of the Au–Ag–Au CSS nanoparticle synthesis corresponds to the smoothening of the nanoparticle surface, which is consistent with the TEM, extinction, and Mie theory results discussed previously. In addition to the SHG peak centered at 400 nm, there is also a very broad signal sloping upward toward longer wavelengths after the first addition of reducing agents, corresponding to two-photon fluorescence.^41,78^ The highest TPF signal intensity occurs during the first addition, as seen in Figure 4, which again correlates to the early stage urchin-like Au–Ag–Au CSS nanoparticle surface. The intensity of the TPF signal decreases after the second addition, due to the smoothening of the nanoparticle surface as observed in the TEM images, in situ extinction spectra, and in situ SHG spectra. The TPF signal is extremely low after the third and fourth additions, which is attributed to the further smoothening of the nanoparticle surface and the reduction of plasmonic hotspots.

**Figure 4:**
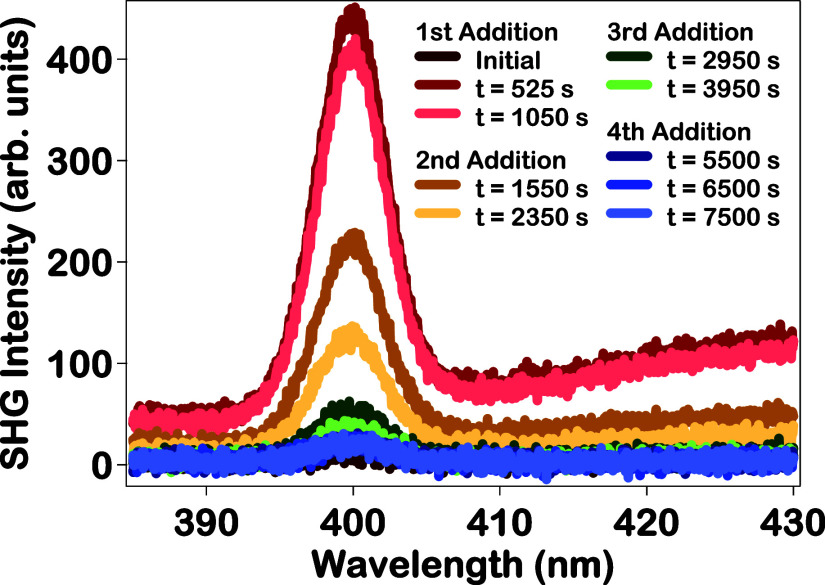
In situ SHG intensity spectra of Au–Ag–Au CSS nanoparticles at various reaction times during the stepwise four additions of the synthesis.

The time-dependent surface-sensitive SHG signal allows for the investigation of the colloidal plasmonic nanoparticle surface in real time^42–45^ to provide complementary information for understanding the nanoparticle growth mechanisms occurring during the Au–Ag–Au CSS nanoparticle synthesis. For direct comparison of the in situ SHG signals, it is necessary to remove the influence of the linear extinction values at 800 and 400 nm, which are primarily from the scattering and absorption by the nanoparticles in solution.^79^ The corrected SHG signal intensity *I*_SHG Corrected_ is obtained using the equation *I*_SHG Corrected_ = *I*_SHG Exp_·10^(ε_800_+(1/2)ε_400_)^ where *I*_SHG Exp_ is the experimental SHG signal intensity and ε_800_ and ε_400_ are the extinction values at 800 and 400 nm, respectively. The SHG electric field *E*_SHG_ is calculated by taking the square root of the integrated corrected SHG signal, given by $E_{\mathrm{SHG}}=\sqrt{I_{\mathrm{SHG}\;\mathrm{Corrected}}}$ . The SHG electric field time trace is shown in Figure 5 along with the corresponding fit for each addition. After the first addition of reducing agents, there is an initial abrupt increase followed by a more gradual decrease which continues until an equilibrium is reached. After each subsequent addition of chloroauric acid and reducing agents, the same trend in the SHG electric field is seen, with an initial rapid increase, a rapid decrease, and then a period of more gradual decrease until reaching an equilibrium. The significant increase in SHG electric field is attributed to the formation of an urchin-like Au–Ag–Au CSS nanoparticle surface, and the decrease corresponds to the smoothening of the nanoparticle surface. The SHG growth lifetimes are determined by fitting the slower time-dependent change with a single-exponential function, given by *E*_SHG_ = *A*_SHG_e^–*t*/τ_SHG_^ + *B*_SHG_ where *A*_SHG_ is the SHG amplitude, τ_SHG_ is the SHG growth lifetime, *B*_SHG_ is the offset SHG electric field, and *t* is the reaction time after the addition of reducing agents. The SHG growth lifetimes determined for the first, second, third, and fourth additions are 126 ± 3, 620 ± 2, 681 ± 7, and 2130 ± 41 s, respectively, as listed in Table 1. The SHG growth lifetimes indicate that longer times are needed for reaching equilibrium surface morphologies as the overall CSS nanoparticle size increases. Separate SHG electric field time traces with corresponding fits are given in Figure S14 and these fit parameters for each addition are tabulated in Table S5 in the Supporting Information.

**Figure 5:**
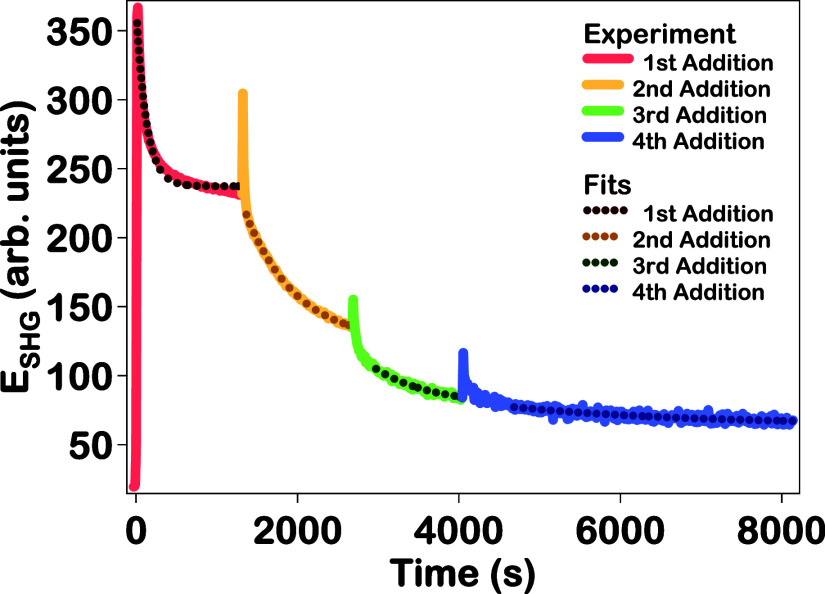
SHG electric field as a function of time for the four stepwise additions of chloroauric acid and reducing agents during the synthesis of Au–Ag–Au CSS nanoparticles (solid lines), along with the corresponding exponential fits (dotted lines).

For additional comparisons to the in situ SHG and extinction spectroscopy results, the in situ TPF spectroscopy is also studied for the Au–Ag–Au CSS nanoparticle synthesis. The corrected TPF signal intensity time trace is shown in Figure 6 along with the corresponding fit of each addition. The TPF signal intensity is corrected to account for the time-dependent linear extinction response, in a similar manner as previously explained for SHG. However, the TPF correction is done with extinction values at 800 and 420 nm, ε_800_ and ε_420_, respectively, to account for the different center wavelength of the TPF, given by the equation, *I*_TPF Corrected_ = *I*_TPF Exp_·10^(ε_800_+(1/2)ε_420_)^ where *I*_TPF Exp_ is the experimental TPF intensity. The corrected TPF intensity *I*_TPF Corrected_ for the first addition initially increases abruptly, then rapidly decreases, followed by a period of more gradual decrease until reaching an equilibrium. After the second addition, the TPF intensity first rapidly decreases, then undergoes a more gradual time-dependent decrease until reaching a new equilibrium of lower TPF signal. These trends mirror those observed for the SHG electric field after the first and second additions. After the third addition, there is another initial decrease followed by a more gradual decrease and equilibrium. Finally, after the fourth addition, the TPF intensity gradually decreases again to reach a final state of equilibrium. Here the formation of plasmonic hotspots during the early growth stages are again associated with an increase of TPF intensity. The relationship between the corrected in situ TPF and SHG intensities during the Au–Ag–Au CSS nanoparticle synthesis is shown by the ratio *I*_TPF_/*I*_SHG_ as a function of the reaction time in Figure S15 of the Supporting Information. The TPF growth lifetimes are also determined by fitting the slower time-dependent change with a single-exponential function, given by *I*_TPF_ = *A*_TPF_e^–*t*/τ_TPF_^ + *B*_TPF_ where *A*_TPF_ is the TPF amplitude, τ_TPF_ is the TPF growth lifetime, and *B*_TPF_ is the offset TPF signal. The TPF growth lifetimes determined for the first, second, and third additions are 94 ± 2, 612 ± 11, and 276 ± 44 s, respectively, as listed in Table 1. The TPF signal during the third and fourth additions is still present but is very low causing higher noise in these measurements. The time-dependent TPF signal for the fourth addition is more accurately represented with a linear fit and therefore no TPF growth lifetime is obtained for this addition. Interestingly, the TPF growth lifetime is lower for the third addition than for the second addition, following a different trend than the SHG growth lifetimes. While the SHG signal is dominated by the nanoparticle surface, the TPF signal is likely caused by a more complicated mixture of surface and bulk spectroscopic effects, where more experimental and theoretical research is needed to better understand these in situ TPF results. Separate TPF intensity time traces with corresponding fits and fit parameters for each addition are given in Figure S15 and Table S6, respectively, in the Supporting Information.

**Figure 6:**
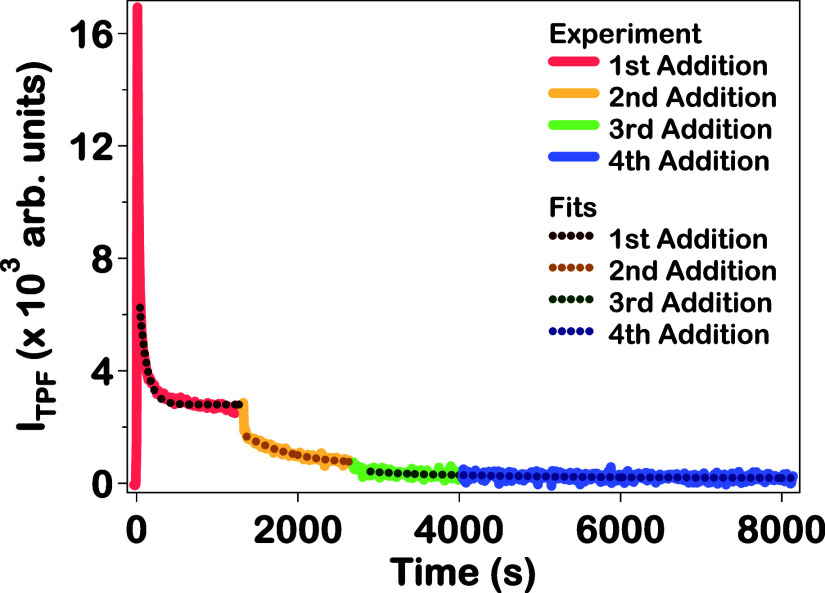
TPF signal intensity as a function of time for the four stepwise additions of chloroauric acid and reducing agents during the synthesis of Au–Ag–Au CSS nanoparticles (solid lines), along with the corresponding fits (dotted lines).

A full comparison of the time-dependent extinction, SHG, and TPF results provides an important context for understanding these different spectroscopic techniques for investigating the plasmonic Au–Ag–Au CSS nanoparticle growth dynamics. The extinction and SHG growth lifetimes are dominated by the bulk nanomaterial and surface of the Au–Ag–Au CSS nanoparticles, respectively, while TPF studies provide complementary information.^41^ The extinction and SHG growth lifetime values increase stepwise with each addition, as seen in Table 1, in agreement with previous studies showing that nanomaterial shell reactions generally take longer for larger nanoparticle sizes.^19,40,41^ However, the SHG growth lifetimes here are considerably longer than the corresponding extinction growth lifetimes, indicating that that time needed to reach an equilibrium outer shell thickness is significantly shorter than the time needed for reaching an equilibrium surface morphology for each addition. The TPF growth lifetimes follow the same trend for the first two additions but decrease with the third addition. For each addition, TPF growth lifetimes are intermediate between the corresponding extinction and SHG growth lifetimes. Also, the TPF signals decrease with each addition, mirroring the SHG signal, suggesting that TPF is enhanced by the plasmonic hotspots present in the urchin-like nanoparticles at early stages of the outer gold shell growth. However, more work is needed, including both experimental and theoretical research, to better understand the TPF process in plasmonic nanomaterials and its dependence on size, composition, surface morphology, and the nanoparticle growth dynamics.

The surface morphology of the final Au–Ag–Au CSS nanoparticles after the fourth addition is further analyzed after washing this colloidal sample twice by centrifugation and redispersion in a clean 135 μM sodium citrate aqueous solution to remove excess salts and reactants. As previously mentioned, small features are observed in the TEM images of the Au–Ag–Au CSS nanoparticles after the third and fourth additions which cause distortions of the nanoparticle surface morphology, as seen in Figures 1 and S7, S8, and are attributed to an excess of reactants and reducing agents in solutions that form small crystals during the sample drying process. After washing, the final Au–Ag–Au CSS nanoparticles are observed to be relatively spherical, smooth, and monodisperse, as shown in the TEM image of Figure 7a, with an average size of 72 ± 19 nm. The experimental and Mie theory calculated extinction spectra of these final Au–Ag–Au CSS nanoparticles after washing are shown in Figure 7b, with a plasmon peak centered at 550 nm compared to the theoretical peak centered at 552 nm. The experimental and theoretical extinction spectra in Figure 7b are in excellent agreement both in terms of plasmon peak wavelength and spectral width, demonstrating a smooth surface morphology and a spherical, concentric CSS nanoarchitecture. Additional characterizations of the final washed CSS nanoparticles are also included in the Supporting Information.

**Figure 7:**
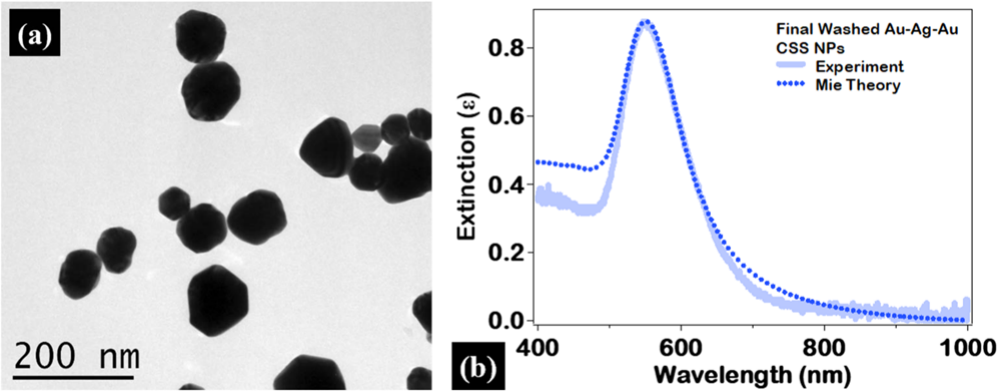
(a) Representative TEM image of the final Au–Ag–Au CSS nanoparticles after washing twice with an average size of 72 ± 19 nm. (b) Extinction spectrum of the final Au–Ag–Au CSS nanoparticles after washing (solid line) along with the corresponding Mie theory results (dotted line). Excellent agreement between the experimental and theoretical extinction spectrum demonstrates a smooth final CSS nanoparticle surface.

This study is the first to our knowledge to use this combined linear and nonlinear spectroscopy approach to study core–shell–shell nanoparticle growth dynamics. The seed-mediated synthesis method and in situ spectroscopy used here follow previous studies on Ag–Au CS nanoparticles^19,69^ for comparison. The results of this work are consistent with our previous studies, demonstrating that this technique is transferable to core–shell–shell nanoparticles and more sophisticated nanoarchitectures for greater size-dependent plasmonic control. The excellent agreement between the experimental extinction spectra and Mie theory calculations further verifies the final Au–Ag–Au CSS nanoparticle results. Additionally, the final size of these Au–Ag–Au CSS nanoparticles are in the ideal range for nanomedicine applications.^4,22,33^ The general observation of multiple additions needed of chloroauric acid and reducing agents for a smooth final shell surface morphology, as with the Ag–Au CS nanoparticles,^19,69^ contrasts with our work on seed-mediated Au and Au–Ag CS nanoparticles,^30,40^ where only one addition of reactants is needed, highlighting the different nanomaterial chemistries. Future work will also determine whether the laser itself causes any significant changes to these plasmonic nanoparticle size or shape distributions. Overall, this research lays the groundwork for additional systematic studies on the role of nanomaterial reactants and their relative concentrations resulting in the final nanoparticle dimensions, surface morphologies, and associated growth dynamics to better control and optimize plasmonic nanomaterials for different targeted applications.

## Conclusions

The stepwise, seed-mediated synthesis of colloidal Au–Ag–Au CSS nanoparticles are studied using a combination of in situ SHG and extinction spectroscopy with Mie theory calculations and TEM imaging. The Au–Ag–Au CSS nanoparticle synthesis consists of four additions of chloroauric acid and the reducing agents, sodium citrate and hydroquinone, to grow an outer gold shell on top of spherical Au–Ag CS nanoparticles with an average core size of 13 nm and an average CS diameter of 21 nm. After the first addition of chloroauric acid and reducing agents, the Au–Ag–Au CSS nanoparticles have an average size of 38 nm and a bumpy, urchin-like surface morphology. With each subsequent addition, the Au–Ag–Au CSS nanoparticles become larger, smoother, and more uniform, while the plasmon peak in the extinction spectra becomes narrower and more blue-shifted with higher peak intensities. Additionally, the deviations between the experimental extinction spectra and the Mie theory calculations decrease as the surface becomes smoother approaching a more ideal CSS architecture, reaching excellent agreement after the fourth addition, where the CSS nanoparticle has a final average diameter of 72 nm. The measured extinction growth lifetimes, which characterize the time needed to reach an equilibrium shell thickness, are shown to increase with each sequential addition of reactants. The stepwise decrease in intensity of SHG and TPF directly tracks with the surface of the Au–Ag–Au CSS nanoparticles becoming smoother. The measured SHG growth lifetimes, which describe the time needed to reach an equilibrium surface morphology, also increase for each sequential addition but are significantly longer than the corresponding extinction values, highlighting the different nanomaterial bulk versus surface spectroscopic sensitivities. The combined approach of Mie theory and TEM measurements with in situ extinction, SHG, and TPF spectroscopies used here provide a comprehensive analysis for understanding the growth dynamics occurring during the Au–Ag–Au CSS nanoparticle synthesis. Additionally, the results of this work are generally consistent with those obtained in our previous studies, demonstrating that this combined characterization technique is transferable to the study of more sophisticated nanoarchitectures for tailoring enhanced plasmonic nanoengineering toward potential nanomedicine applications.

## Supplementary Material



## Data Availability

The data that support the findings of this study are available from the corresponding author upon reasonable request.

## Notes

Characterization of gold seeds and Au–Ag CS nanoparticles, additional TEM images of Au–Ag–Au CSS nanoparticles, size distribution histograms, additional in situ extinction spectra, and individual extinction, SHG, and TPF time traces with corresponding tabulated fit parameters (PDF)
