# Supplementary material for: In Situ Second Harmonic Generation and Extinction Spectroscopy for Studying Colloidal Gold–Silver–Gold Core–Shell–Shell Nanoparticle Growth Dynamics
Source: J Phys Chem C Nanomater Interfaces. 2025 Jun 13;129(26):12033–42. doi: 10.1021/acs.jpcc.5c02596 (PMC12235635; doi:10.1021/acs.jpcc.5c02596)
Supplement: Supplementary file 1 [file jp5c02596_si_001.pdf]

Supporting Information for

In Situ Second Harmonic Generation and Extinction Spectroscopy for Studying Colloidal Gold-Silver-Gold Core-Shell-Shell Nanoparticle Growth Dynamics

Stena C. Peterson, Daniel A. Babayode, Christopher P. Reso, and Louis H. Haber*

Department of Chemistry, Louisiana State University, Baton Rouge, Louisiana 70803, USA.

*Corresponding author's email: lhaber@lsu.edu

Characterizations of gold nanoparticle seeds and gold-silver CS nanoparticles

A representative transmission electron microscopy (TEM) image, size distribution histogram, and extinction spectrum for the gold nanoparticle seeds are shown in Figure S1. The Au seed size distribution histogram is obtained from TEM diameter measurements of over 150 nanoparticles, where the average size is obtained from a logarithmic-normal fit. The experimental extinction spectrum of the Au seeds shows excellent agreement with the result calculated from Mie theory using the Au seed size distribution. Additional representative TEM images of the Au seeds are shown in Figure S2.

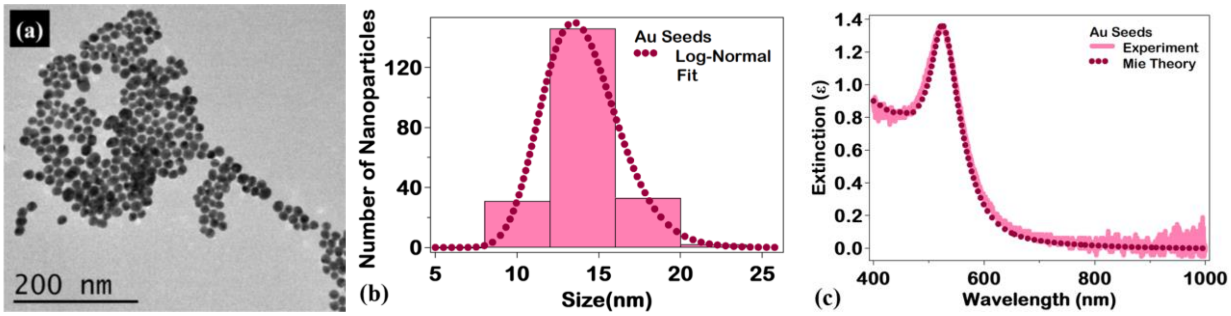

Figure S1. (a) Representative TEM image of the Au seeds. (b) Size distribution histogram for the Au seeds with corresponding log-normal fit (dotted line) giving the average diameter of 13.2 ± 1.9 nm. (c) Experimental extinction spectrum (solid line) along with the corresponding Mie theory results (dotted line) for the Au seeds.

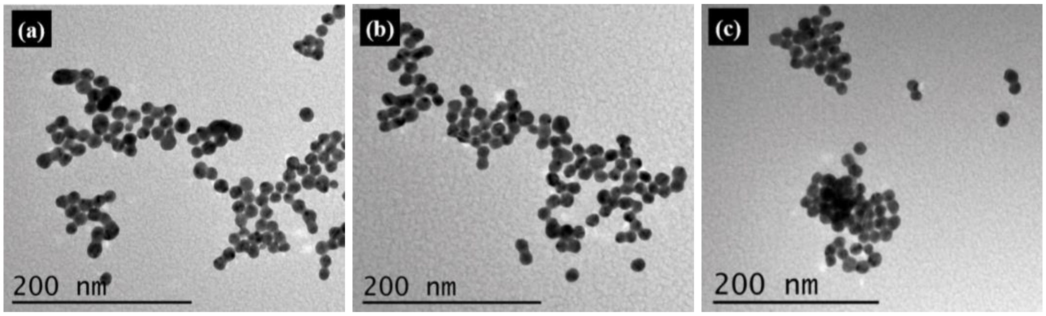

Figure S2. Additional TEM images of the Au seeds used to synthesize the Au-Ag CS nanoparticles.

Gold-silver core-shell (Au-Ag CS) nanoparticles are prepared using a seed-mediated growth procedure from the Au seeds shown in Figures S1 and S2. Figure S3 shows a representative TEM image, size distribution histogram, and extinction spectrum for the Au-Ag CS nanoparticles. The experimental extinction spectrum of the Au-Ag CS nanoparticles shows general agreement with the result calculated from Mie theory. Additional representative TEM images of the Au-Ag CS nanoparticles are shown in Figure S4. The Au-Ag CS nanoparticles have an average diameter of 20.8 ± 6.8 nm determined from the logarithmic-normal fit of the size distribution data, giving an average silver shell thickness of 3.8 nm.

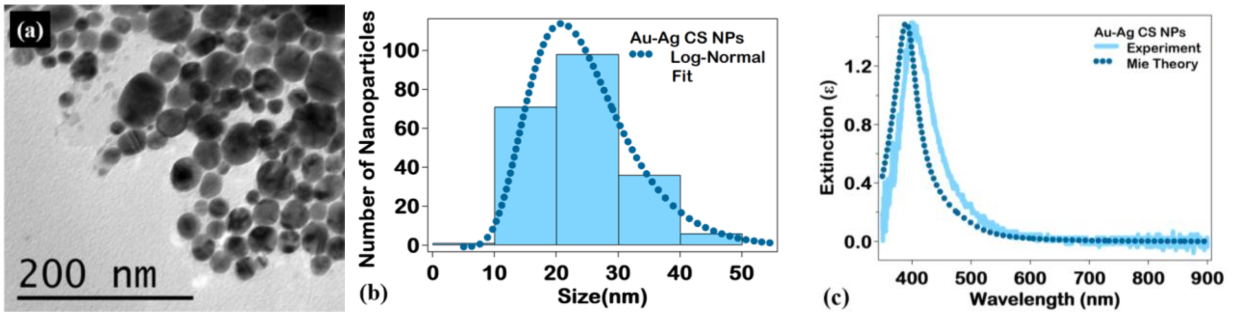

Figure S3. (a) Representative TEM image of the Au-Ag CS nanoparticles prepared using the precursor Au seeds. (b) Size distribution histogram for the Au-Ag CS nanoparticles with corresponding log-normal fit (dotted line) giving an average size of 20.8 ± 6.8 nm. (c) Experimental extinction spectrum (solid line) along with the corresponding Mie theory results (dotted line) for the Au-Ag CS nanoparticles.

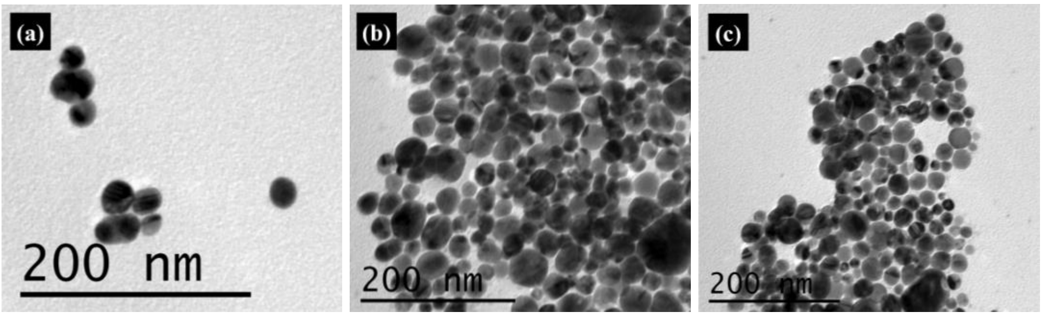

Figure S4. Additional TEM images of the Au-Ag CS nanoparticles used for the stepwise synthesis of the Au-Ag-Au CSS nanoparticles.

These Au-Ag CS nanoparticles are used for the synthesis of gold-silver-gold core-shell-shell (Au-Ag-Au CSS) nanoparticles which is monitored in real time using *in situ* second harmonic generation (SHG) and extinction spectroscopy to study the growth processes involved, as explained in the paper. Additional representative TEM images of the Au-Ag-Au CSS nanoparticles after the first, second, third, and fourth additions of chloroauric acid and reducing agents are shown in Figures S5, S6, S7, and S8, respectively. Additional TEM images of the washed final Au-Ag-Au CSS nanoparticles after the fourth addition are shown in Figure S9. The size distribution histograms for the four additions and the washed final nanoparticles are displayed in Figure S10. The average sizes determined from the logarithmic normal fit, indicated by the dotted line in each

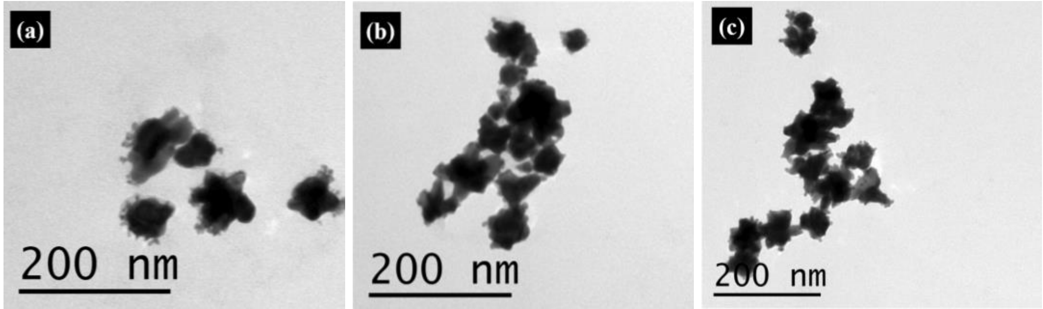

Figure S5. Additional TEM images of Au-Ag-Au CSS nanoparticles after the first addition of chloroauric acid and reducing agents using the precursor Au-Ag CS nanoparticles.

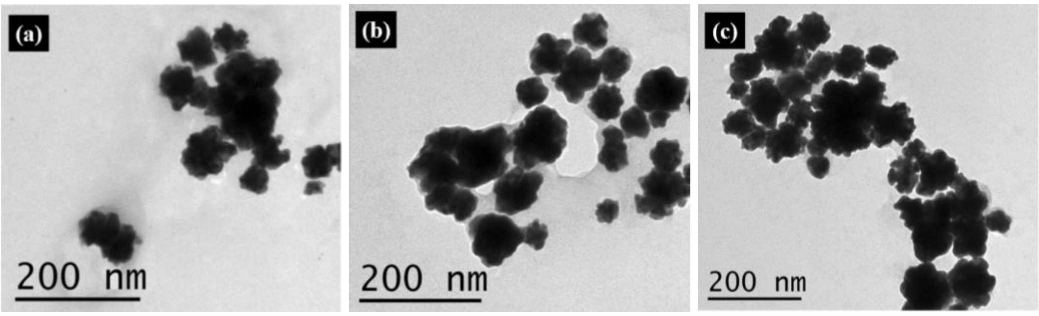

Figure S6. Additional TEM images of Au-Ag-Au CSS nanoparticles after the second addition of chloroauric acid and reducing agents.

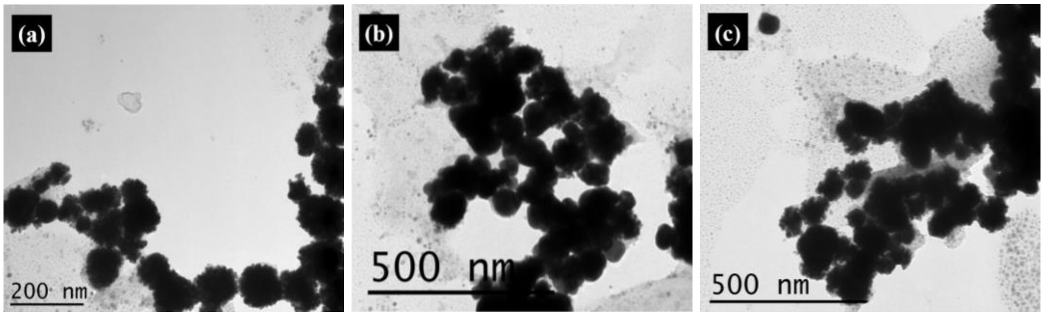

Figure S7. Additional TEM images of Au-Ag-Au CSS nanoparticles after the third addition of chloroauric acid and reducing agents.

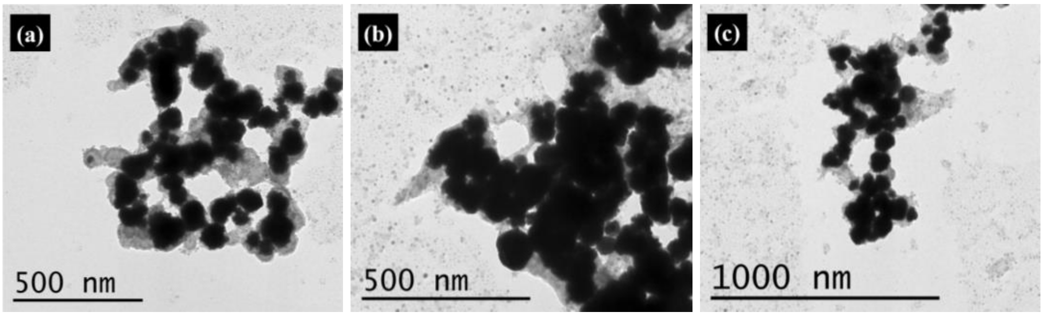

Figure S8. Additional TEM images of Au-Ag-Au CSS nanoparticles after the fourth addition of chloroauric acid and reducing agents.

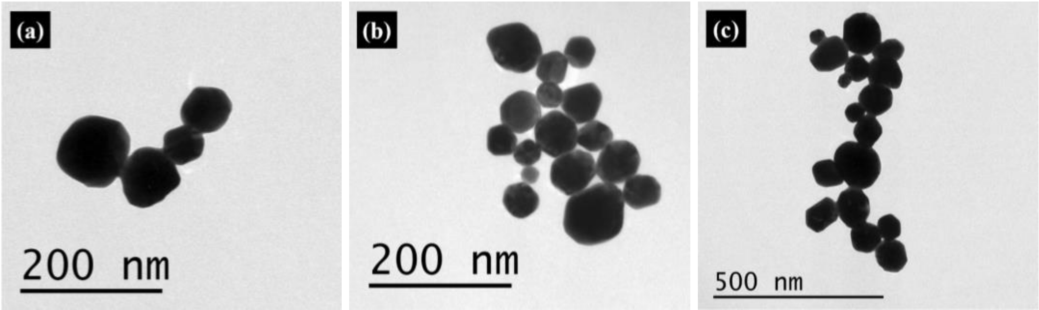

Figure S9. Additional TEM images of Au-Ag-Au CSS nanoparticles after washing the final nanoparticles.

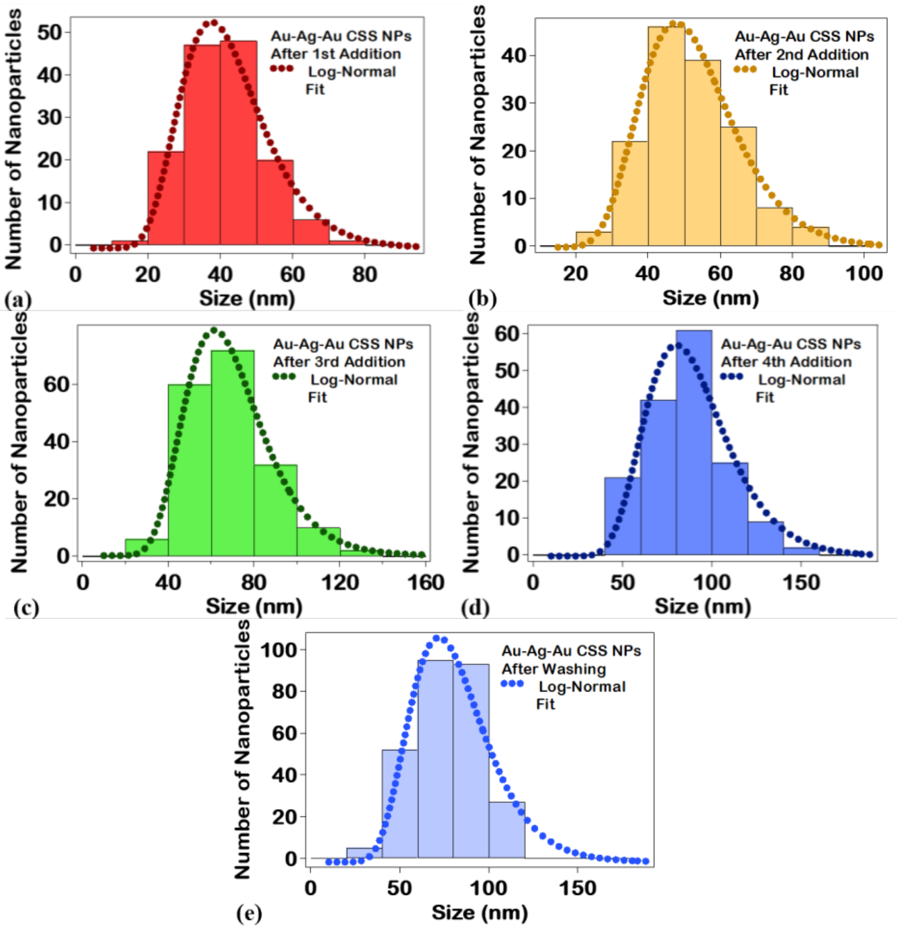

Figure S10. Size distribution histograms for Au-Ag-Au CSS nanoparticles after the (a) first addition, (b) second addition, (c) third addition, (d) fourth addition, and (e) washing, with corresponding log-normal fits (dotted lines).

histogram, after the first, second, third, and fourth additions of chloroauric acid and reducing agents and the washed final Au-Ag-Au CSS nanoparticles are 37.5 ± 10.7 nm, 47.8 ± 12.3 nm, 61.5 ± 17.4 nm, 80.1 ± 21.7 nm, and 71.9 ± 19.0 nm, respectively. The added significant digits for the CSS dimensions and standard deviations listed in the Supporting Information are used for the Mie theory calculations for improved accuracy.

Additional details on *in situ* extinction, SHG, and TPF results

The temporal *in situ* extinction spectra of Au-Ag-Au CSS nanoparticles, shown in Figure S11, depict the change in the extinction spectra at various reaction times after each addition of

chloroauric acid and reducing agents. A general trend of a more red-shifted, broader plasmonic peak that blue-shifts and narrows as the final gold shell formation occurs is observed for all four additions, reaching stable plasmonic spectra for each addition.

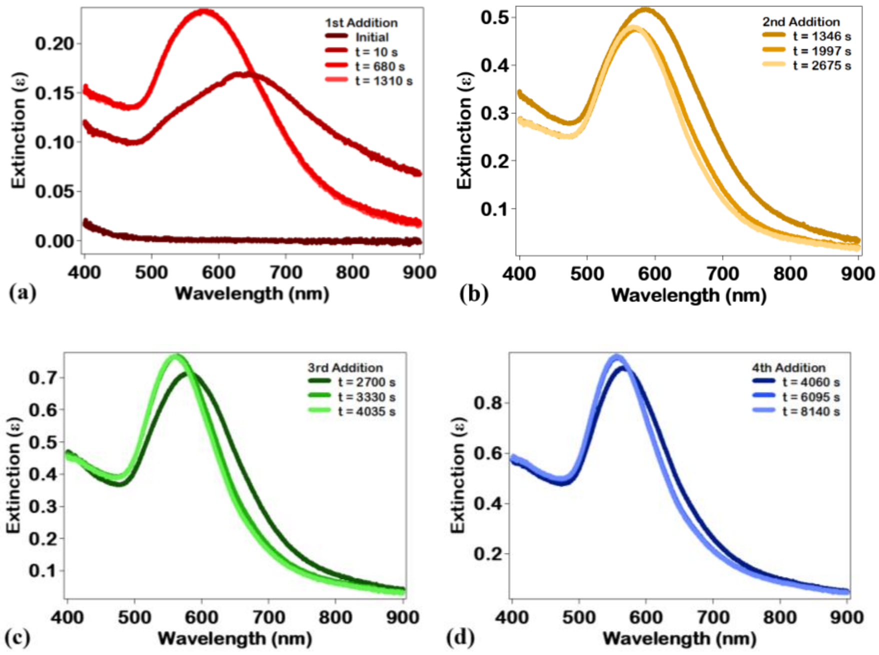

Figure S11. *In situ* extinction spectra of Au-Ag-Au CSS nanoparticles at various times during the stepwise synthesis in the (a) first, (b) second, (c) third, and (d) fourth additions of chloroauric acid and reducing agents.

Individual time traces of the extinction peak maximum values and their corresponding fits during the formation of the outer gold shell onto the Au-Ag CS nanoparticles are shown in Figure S12. The extinction amplitudes A_{ext}, growth lifetimes τ_{ext}, and offsets B_{ext} for the first, second, and third additions determined from the corresponding fits, as seen in Figure 3 and Figure S12, are tabulated in Table S1. Interestingly, the second addition shows a positive A_{ext} from the downward decay in extinction peak maximum, which is different than the first and third additions. This might be caused by a confluence of competing processes occurring as the nanoparticle surface becomes smoother while the shell thickness also increases for this specific size distribution. Instead of an exponential trend as seen for the first three additions, the fourth addition extinction peak maximum

time trace follows a linear trend given by $\varepsilon(t) = at + b$, in which the fit parameters are determined to be and a = (1.98 ± 0.01) × 10^{-6} ε/s and b = 0.969 ± 0.001 ε.

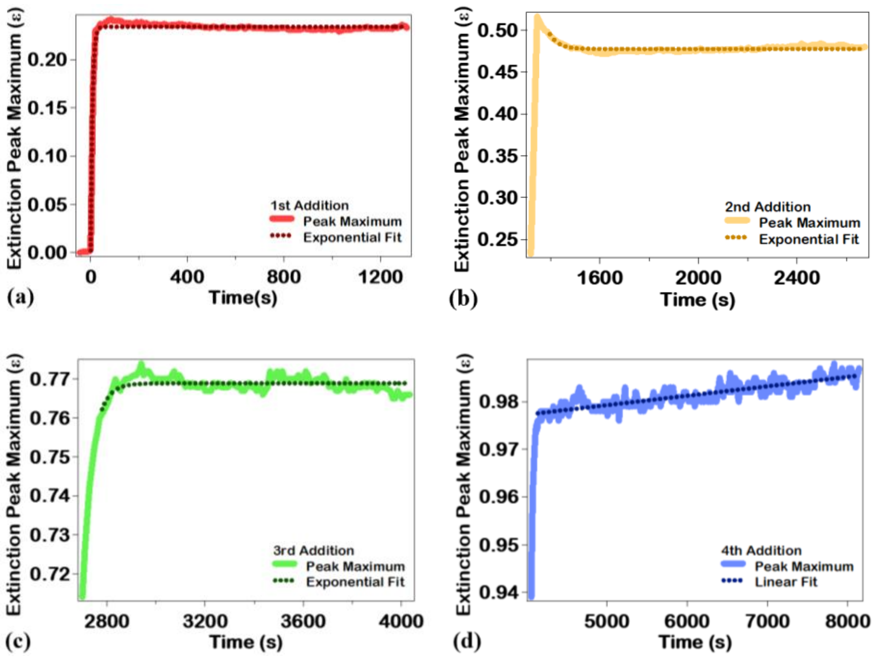

Figure S12. Extinction peak maximum as a function of time for the (a) first, (b) second, (c) third, and (d) fourth addition of the four stepwise additions of chloroauric acid and reducing agents during the synthesis of Au-Ag-Au CSS nanoparticles, shown by the solid lines, along with the corresponding exponential fits for the first three additions and the corresponding linear fit for the fourth addition, shown by the dotted lines.

Table S1. Fitting parameters obtained from the *in situ* extinction peak maximum as a function of time for the first three stepwise additions of chloroauric acid and reducing agents.

Addition	A_{ext}	τ_{ext}	B_{ext}
First	-0.232 ± 0.003	7.0 ± 0.2 s	0.234 ± 0.001
Second	0.177 ± 0.019	37.5 ± 1.5 s	0.477 ± 0.001
Third	-0.089 ± 0.018	40.4 ± 0.1 s	0.769 ± 0.001

A customized data analysis program is used for the analysis of the SHG and TPF data, as described in our previous work.[1] Briefly, the background-subtracted SHG spectra is taken as a function of time, from repeating iterations of 5 spectra with the laser unblocked minus 5 spectra with the laser blocked at 1.0 s acquisition times. The SHG signal is separated from the overlapping TPF signal by analysis of different spectra regions. The signal from 393 nm to 407 nm is first

integrated, which corresponds to the overall signal of SHG and TPF over this range. The TPF signal over this range is determined by a linear fit of the signal intensities from 388 nm to 393 nm and from 407 nm to 412 nm, which does not include SHG. Then, that linear fit is used to calculate the integrated TPF for the range of 393 nm to 407 nm, which is subtracted from the overall signal in this range to determine the experimental integrated SHG intensity. Figure 4 shows representative background-subtracted SHG intensity spectra along with overlapping TPF signals at different reaction times. These time-dependent experimental SHG intensity values are then corrected using the time-dependent linear extinction values, as explained in the paper. The experimental time-dependent TPF signals are determined by integrating the time-dependent background-subtracted spectra from 410 nm to 424 nm. Both the SHG intensities and the TPF intensities correspond to different 300-pixel widths on the same high-sensitivity spectroscopy CCD detector for direct comparisons.

The SHG and TPF corrections rely on the careful measurements of the *in situ* extinction values at 800, 400, and 420 nm. These values are shown in Figure S13 along with the combination of linear fits given by ε_i (t) = $a_i\ t + \ b_i$ and/or single exponential fits given and ε_i (t) = $A_i\ e^{-t/\tau_i} + B_i$ for each wavelength i = 800, 400, and 420 nm, respectively. The first addition follows a linear fit until t = 20 s, then an exponential fit for the remaining time of the addition. The second, third, and fourth additions all use exponential fits, except for the fourth addition ε_{420} time trace which uses a linear fit. The extinction values used for the correction of the experimental SHG and TPF intensities are then calculated at the appropriate reaction times using the fits and fit parameters determined. These extinction fit values are used for the SHG and TPF corrections for better accuracy. The fits and fit parameters determined for the extinction data at 800 nm, 400 nm, and 420 nm are summarized in Tables S2, S3, and S4, respectively.

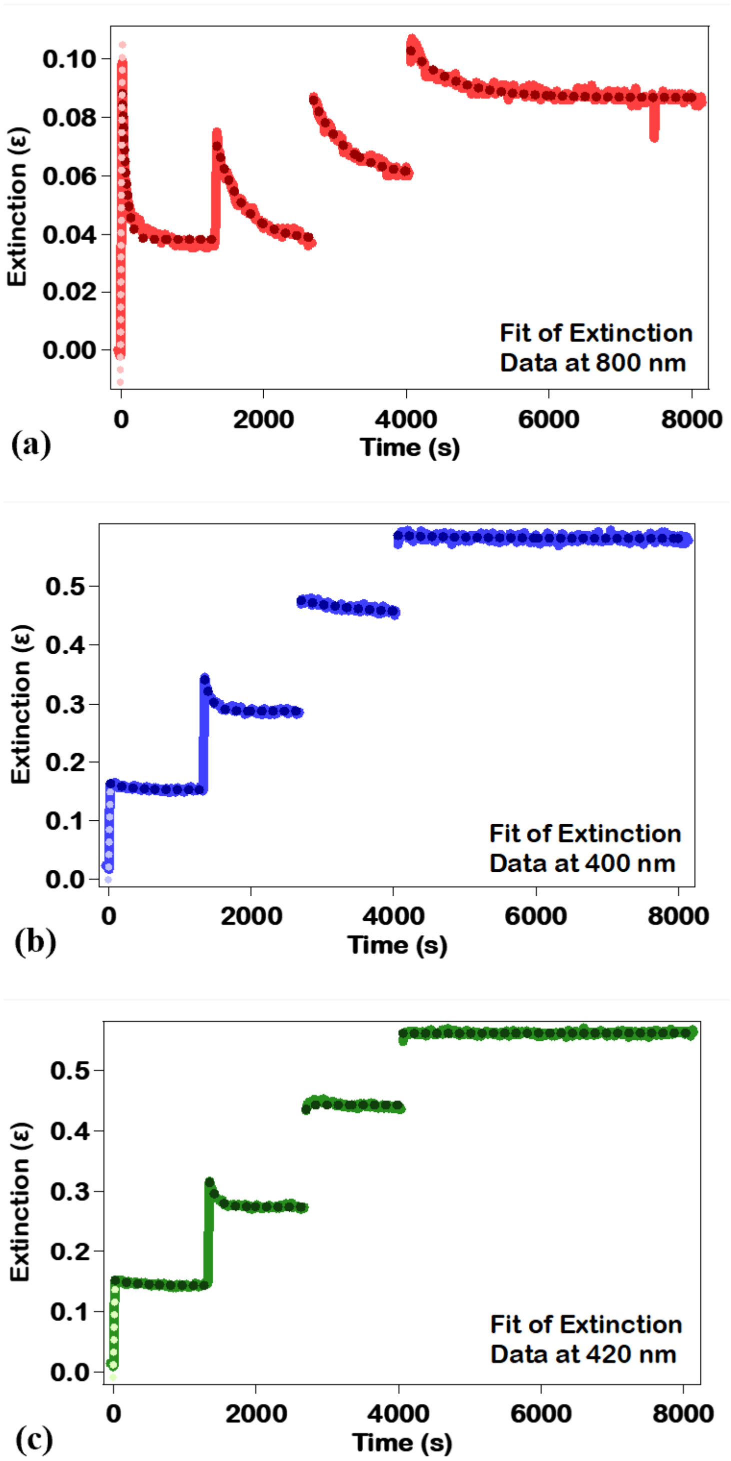

Figure S13. *In situ* extinction data at (a) 800 nm, (b) 400 nm, and (c) 420 nm as a function of time, given by the solid red, blue, and green lines, respectively. The corresponding fits of the extinction data are shown by the dotted lines for more accurate corrections to the SHG and TPF signals.

Table S2. Fit parameters determined for the extinction data measurements at 800 nm as a function of time, seen in Figure S13(a), which are then used for the correction of the SHG and TPF intensities.

Fit of Extinction Data at 800 nm			
Addition	Fit	Fit Parameters	
1st	Linear	a	0.004 ± 0.001
		b	0.029 ± 0.012
	Single exponential	A	0.069 ± 0.002
		τ	61.4 ± 2.0
		B	0.038 ± 0.0002
2nd	Single exponential	A	1.263 ± 0.013
		τ	366.7 ± 0.9
		B	0.038 ± 0.0001
3rd	Single exponential	A	9.568 ± 0.100
		τ	456.5 ± 0.7
		B	0.060 ± 0.0001
4th	Single exponential	A	14.015 ± 0.276
		τ	599.1 ± 1.6
		B	0.087 ± 0.0001

Table S3. Fit parameters determined for the extinction data measurements at 400 nm as a function of time, seen in Figure S13(b), which are then used for the correction of the SHG intensities.

Fit of Extinction Data at 400 nm			
Addition	Fit	Fit Parameters	
1st	Linear	a	0.005 ± 0.001
		b	0.053 ± 0.012
	Single exponential	A	0.011 ± 0.001
		τ	266.2 ± 22.0
		B	0.153 ± 0.0002
2nd	Single exponential	A	0.288 ± 0.0003
		τ	40119 ± 997
		B	0.288 ± 0.0003
3rd	Single exponential	A	0.452 ± 0.0003
		τ	0.451 ± 0.011
		B	0.452 ± 0.0003
4th	Single exponential	A	0.581 ± 0.0002
		τ	0.229 ± 0.029
		B	0.581 ± 0.0002

Table S4. Fit parameters determined for the extinction data measurements at 420 nm as a function of time, seen in Figure S13(c), which are then used for the correction of the TPF intensities.

Fit of Extinction Data at 420 nm			
Addition	Fit	Fit Parameters	
1st	Linear	a	0.005 ± 0.001
		b	0.044 ± 0.012
	Single exponential	A	0.009 ± 0.0005
		τ	324.3 ± 22.8
		B	0.143 ± 0.0002
2nd	Single exponential	A	15363 ± 309
		τ	104.7 ± 0.2
		B	0.275 ± 0.0002
3rd	Single exponential	A	-8.32E68 ± 3.97E68
		τ	16.5 ± 0.05
		B	0.443 ± 0.0004
4th	Linear	a	1.25E-07 ± 2.25E-08
		b	0.561 ± 0.0001

Individual SHG electric field time traces and their corresponding single exponential fits during the formation of the outer gold shell onto the Au-Ag CS nanoparticles are shown in Figure S14. The SHG amplitudes A_{SHG}, growth lifetimes τ_{SHG}, and offsets B_{SHG} for the first, second, third, and fourth additions determined from their corresponding fits, as seen in Figures 5 and S14, are tabulated in Table S5. Individual time traces of the corrected TPF signal intensity and their corresponding fits during the formation of the outer gold shell onto the Au-Ag CS nanoparticles are shown in Figure S15. The TPF amplitudes A_{TPF}, growth lifetimes τ_{TPF}, and offsets B_{TPF} for the first, second, and third additions determined from their corresponding fits, as seen in Figure 6 and Figure S15, are tabulated in Table S6. The fourth addition TPF time trace is most accurately represented by a linear fit $I_{TPF}\ (t) = at + b$ instead of an exponential fit, where the fit parameters are determined to be a = -0.023 ± 0.002 s^{-1} and b = 268 ± 5.

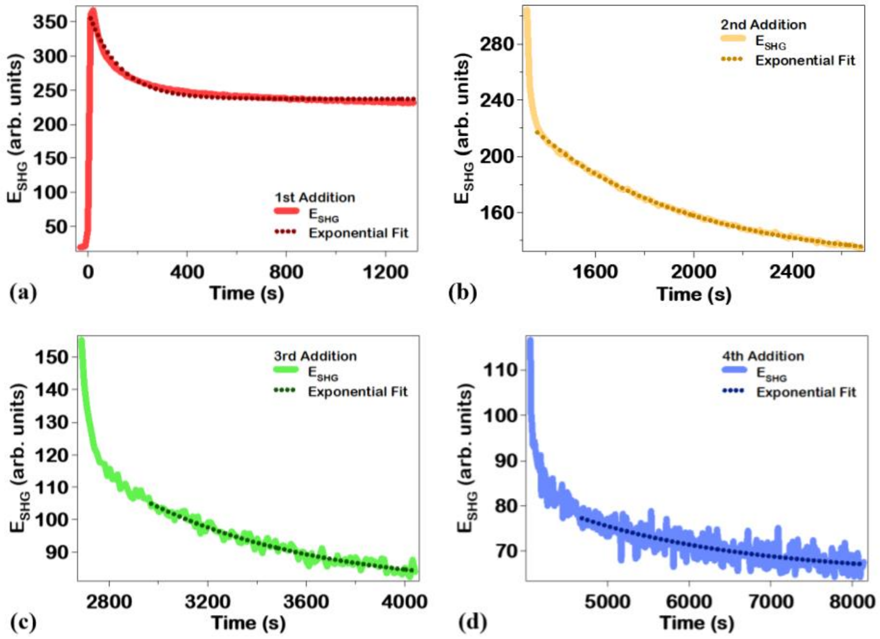

Figure S14. SHG electric field as a function of time for the (a) first, (b) second, (c) third, and (d) fourth additions of chloroauric acid and reducing agents during the synthesis of Au-Ag-Au CSS nanoparticles, shown by the solid lines, along with the corresponding fits for each addition, represented by the dotted lines.

Table S5. Fitting parameters obtained from the *in situ* SHG electric field as a function of time for the four stepwise additions of chloroauric acid and reducing agents.

Addition	A_{SHG}	τ_{SHG}	B_{SHG}
First	129 ± 2	126 ± 3 s	237 ± 0.4
Second	100 ± 0.2	620 ± 2 s	124 ± 0.1
Third	40.4 ± 0.4	681 ± 7 s	78.9 ± 0.1
Fourth	17.0 ± 0.3	2130 ± 41 s	64.6 ± 0.1

In Figure S16, the relationship between the corrected TPF and corrected SHG intensities over the course of the Au-Ag-Au CSS nanoparticle synthesis is shown by the ratio of I_{TPF}/I_{SHG} versus reaction time. A closer look at the I_{TPF}/I_{SHG} ratio as a function of reaction time for each addition is shown in Figure S17. The I_{TPF}/I_{SHG} ratio reaches a peak shortly after the first addition of reducing agents, corresponding to the highly urchin-like surface morphology. The I_{TPF}/I_{SHG} ratio then decreases and remains relatively constant near 0.04 for the majority of the second, third,

and fourth additions, where large noise results from the very low TPF signals. Overall, this work indicates that TPF depends on several factors, including the nanomaterial surface and bulk properties. More experimental and theoretical research is needed to better understand the differences between SHG and TPF in characterizing plasmonic nanoparticle growth dynamics.

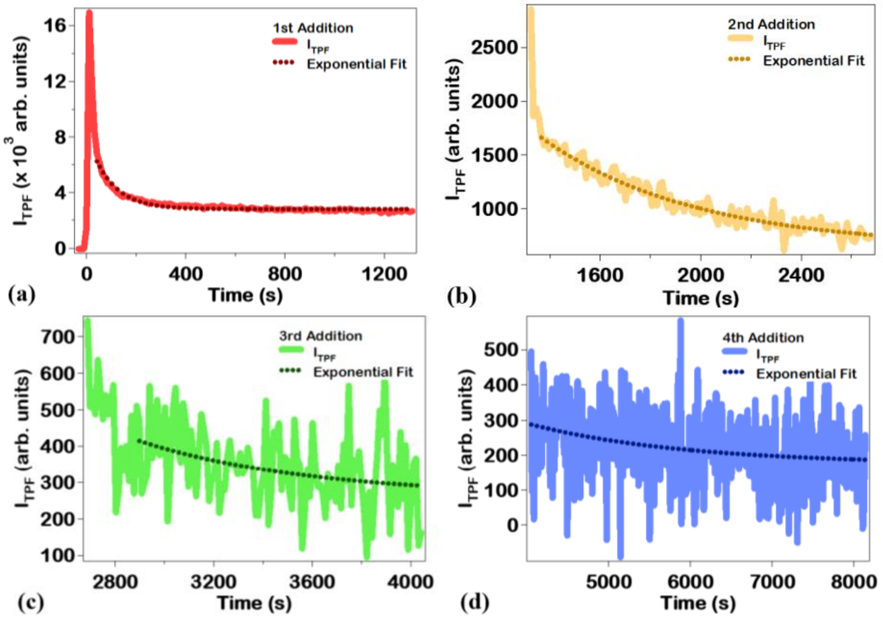

Figure S15. TPF intensity as a function of time for the (a) first, (b) second, (c) third, and (d) fourth additions of chloroauric acid and reducing agents during the stepwise synthesis of Au-Ag-Au CSS nanoparticles, shown by solid lines, along with the corresponding fits for each addition, represented by dotted lines.

Table S6. Fitting parameters obtained from the *in situ* TPF intensity as a function of time for the first three stepwise additions of chloroauric acid and reducing agents.

Addition	A_{TPF}	τ_{TPF}	B_{TPF}
First	5397 ± 103	94 ± 2 s	2799 ± 13
Second	1117 ± 14	612 ± 11 s	638 ± 6
Third	250 ± 29	276 ± 44 s	307 ± 9

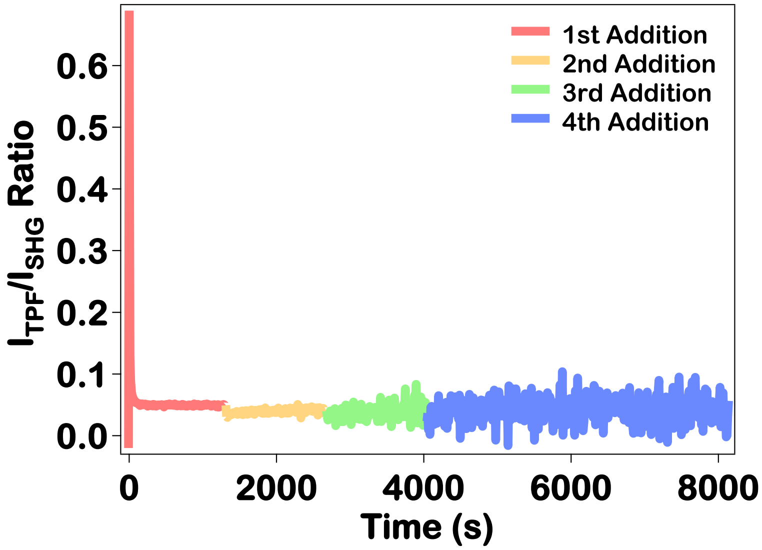

Figure S16. Ratio of TPF intensity to SHG intensity as a function of time for the four stepwise additions of chloroauric acid and reducing agents during the synthesis of Au-Ag-Au CSS nanoparticles.

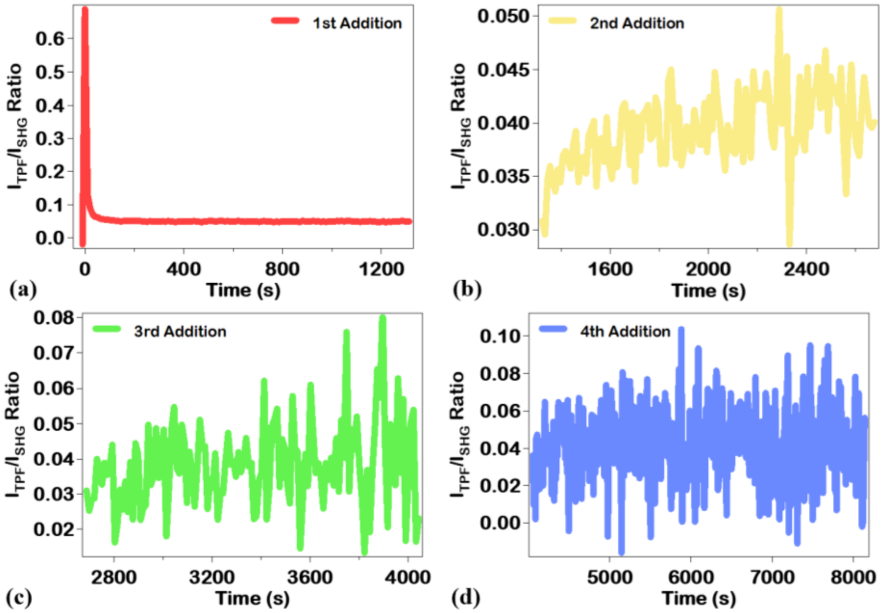

Figure S17. Ratio of TPF intensity to SHG intensity as a function of time for the (a) first, (b) second, (c) third, and (d) fourth additions during the synthesis of Au-Ag-Au CSS nanoparticles.

Reference

(1) Babayode, D. A.; Peterson, S. C.; Haber, L. H. Size-Dependent Growth Dynamics of Silver–Gold Core–Shell Nanoparticles Monitored by in Situ Second Harmonic Generation and Extinction Spectroscopy. *J. Chem. Phys.* **2024**, *161* (8), 084710. https://doi.org/10.1063/5.0217901.
